# C5aR^+^ dendritic cells fine-tune the Peyer’s patch microenvironment to induce antigen-specific CD8^+^ T cells

**DOI:** 10.1038/s41541-023-00720-z

**Published:** 2023-08-14

**Authors:** Sae-Hae Kim, Eun-Hyeon Shim, Doo-Jin Kim, Yong-Suk Jang

**Affiliations:** 1 Department of Molecular Biology and the Institute for Molecular Biology and Genetics, Jeonbuk National University, Jeonju, 54896 South Korea; 2 Innovative Research and Education Center for Integrated Bioactive Materials and the Department of Bioactive Material Sciences, Jeonbuk National University, Jeonju, 54896 South Korea; 3 Infectious Disease Research Center, Korea Research Institute of Bioscience and Biotechnology (KRIBB), Daejeon, South Korea; 4 Department of Bioscience, University of Science and Technology (UST), Daejeon, 34113 South Korea; 5 Department of Biochemistry, Chungnam National University, Daejeon, 34134 South Korea

**Keywords:** Adjuvants, Antigen presentation

## Abstract

The mucosal delivery route is considered ideal for immunization. However, induction of antigen-specific mucosal immunity is difficult due to the tolerogenic environment. Therefore, developing an immunogenic mucosal dendritic cell (DC)-targeting strategy is required. Herein, we investigated the characteristics and immunogenic potential of Peyer’s patch (PP) DCs as an oral vaccination-targeting strategy. Single-cell RNA sequencing analysis of the PP DCs showed that complement C5a receptor- and lysozyme-expressing DCs exhibit increased expression of genes related to chemotaxis. Administration of the Co1 peptide, a C5aR ligand, increased CD8^+^ T cell infiltration and response to the co-delivered model antigen in mice. Furthermore, in the SARS-CoV-2 vaccine model, vaccination with Co1 elicited both systemic and mucosal immunity. Collectively, these findings demonstrate that C5aR signaling in mucosal DCs plays a role in regulating adjuvant activity by modulating the tissue microenvironment.

## Introduction

Protective immunity in the mucosa of various organs, including the lungs and gut, is essential for responding to various emerging infectious agents, including SARS-CoV-2. Protective immunity against infectious agents can be acquired by mucosal vaccination, inducing a broad range of immune responses within not only the mucosa, which is the frontline of pathogen infection throughout the body, but also the systemic compartment^1^. Although few mucosal vaccines are available, most are limited to the live attenuated platform. Mucosal vaccines based on other platforms, including recombinant protein subunits, have not yet been approved. Considering the safety and convenience of vaccination, the development of recombinant protein-based mucosal vaccines is essential.

The most important obstacle to the development of various mucosal vaccine platforms is the tolerogenic mucosal immune environment, which is determined by the characteristics of dendritic cells (DCs) encountering antigens^2^. For example, when mice that were adoptively transferred with OT-II cells were orally gavaged with ovalbumin (OVA), the frequency of forkhead box P3 (FOXP3^+^) OT-II cells in the gut-draining lymph nodes (gLN) was high in the duodenum (the proximal part of the small intestine) and gradually decreased toward the cecum. The frequency of FOXP3^+^ T cells in the gLN correlated with the ratio of tolerogenic (CD103^+^CD11b^-^) to proinflammatory (CD103^+^CD11b^+^) DCs in the duodenum and caecum^3^. CD103^+^CD11b^-^ type-1 conventional DCs (cDC1s) in the lamina propria (LP) drive the generation of regulatory CCR9^+^ FOXP3^+^CD8^+^ T cells against intestinal epithelial cell-derived antigens^4^. This means that delivery of mucosal vaccines to both the duodenal gLN and LP will likely induce immune tolerance to the vaccine. The regulation of T cell immunity by DCs has also been reported in the respiratory mucosa. The default function of cDCs (CD11c^+^MHCII^+^F4/80^-^) localized in the nasal-associated lymphoid tissue (NALT), a lymphoid organ in the submucosa of the nasal passage, is to suppress T cell responses against inhaled antigens. In contrast, monocyte-derived DCs (moDCs, MAR-1^+^CD64^+^), which are recruited in the NALT according to the inflammatory signal following influenza virus infection, induce antigen-specific T cell responses^5^. These findings indicate that the induction of protective immunity via mucosal vaccination is determined by the DCs where mucosal vaccines are delivered.

Unlike LP DCs, Peyer’s patch (PP) DCs are ideal targets for vaccine delivery to orchestrate adaptive immunity. PPs are well-organized gut-associated lymphoid tissues, and PP DCs predominantly induce T cell priming to luminal antigens transported via microfold (M) cells, which are epithelial cells specialized for the uptake of bacteria and particulate antigens^6^. PP DCs consist of cDC1s (CD11c^+^MHCII^+^SIRPa^-^), type-2 cDCs (cDC2s, CD11c^+^MHCII^+^SIRPa^+^BST^-^), lysozyme-expressing monocyte-derived DCs (LysoDCs, CD11c^+^MHCII^+^SIRPa^+^BST^int^), and plasmacytoid DCs (pDCs, CD11c^+^MHCII^+^SIRPa^+^BST^hi^)^7^. Among them, LysoDCs take up pathogens, such as *Salmonella typhimurium* and *Listeria monocytogenes*, by extending their dendrites to transcellular pores in M cells^8,9^. In addition, the function of LysoDCs can be modulated by innate immune stimuli, implying that LysoDCs are attractive targets for vaccination. For instance, stimulation of LysoDCs with the Toll-like receptor 7 (TLR7) ligand not only drives the expression of cytokines (e.g., IL-6 and TNF) but also naïve CD4^+^ T cell priming^10^. In addition, complement C5a-mediated stimulation of LysoDCs expressing the C5a receptor (C5aR^+^ LysoDCs) provokes exogenous antigen-specific CD8^+^ T cell responses by inducing cross-presentation^11^. Given that the ability to cross-present exogenous antigens is essential to induce antigen-specific CD8^+^ T cell responses with recombinant protein antigens, the application of vaccine adjuvants stimulating C5aR^+^ LysoDC is a promising strategy^12^.

Approaches to developing mucosal vaccine adjuvants have focused on potentiating the immune response induced by subunit vaccines using enterotoxin-derived adjuvants or invariant natural killer T cell activators^13,14^. However, studies on mucosal adjuvants targeting PP DCs have been poorly conducted. In this study, we investigated whether C5aR^+^ LysoDC has potential as a target cell for mucosal vaccine adjuvants based on functional analysis of the transcripts expressed in PP DCs. To modulate C5aR^+^ LysoDCs, we used the Co1 peptide among the C5aR ligands as a mucosal vaccine adjuvant. The Co1 peptide was previously reported as a targeting ligand for C5aR on M cells. When the Co1 peptide was applied to the antigen, the antigen-specific immune response increased in various mucosal vaccine models^15,16^. However, the mechanism of the Co1 peptide as a mucosal adjuvant remains to be fully clarified. Therefore, in this study, we aimed to identify the mechanism of Co1 peptide-mediated C5aR signaling by investigating chemotaxis and exogenous antigen cross-presentation in C5aR^+^ LysoDCs. Additionally, the adjuvant ability of the Co1 peptide was verified in a mucosal vaccine model using the S1 domain of the SARS-CoV-2 spike protein.

## Results

### C5aR^+^ LysoDCs are an attractive target for mucosal vaccine delivery

The immune response elicited by mucosal vaccines is determined by the nature of the DCs (effector T cells or regulatory T cell (Treg) priming) encountered at the mucosal immune-inductive site^17^. To identify the target DCs for mucosal vaccine application, we re-analyzed the single-cell RNA sequencing (scRNA-seq) data of 7463 sorted CD11c^hi^MHC^hi^SIRPα^+^ PP cells consisting of cDC2s and monocyte-derived LysoDCs, which are mainly located in the subepithelial dome (SED) (GEO: GSE165040)^11^. Uniform manifold approximation and projection (UMAP) dimensionality reduction analysis showed that the transcriptional profiles of cluster 3 were distinct from those of the other clusters (Supplementary Fig. 1a). The identities of the clusters were analyzed using the SingleR and ImmGen’s reference databases (Supplementary Fig. 1b). Although cluster 3 was defined as macrophages in the SingleR annotation, it did not express classic macrophage markers, including *Fcgr1* (encoding CD64) and *Adgre1* (encoding F4/80). However, the expression of signature genes for LysoDC (*Lyz1* and *Ccr2)* was highly enriched in cluster 3^8^. Consequently, we defined cluster 3 as LysoDCs, but not macrophages, and the others as cDC2s, except for cluster 10, which was thought to be a contamination of non-DC populations.

To identify the vaccine adjuvant targeting cluster, we visualized the expression pattern of the top 10 genes per cluster using heatmaps and dot plots and analyzed their biological functions by Gene-Set Enrichment Analysis using gProfiler, which displays the results as a dot plot (Supplementary Fig. 1c, d, and Supplementary Fig. 2). As reported previously for other tissues, PP cDC2s are heterogeneous. For example, clusters 0 and 5 were identified as activated cDC2s based on the enriched expression of *Ciita* (MHC class II complex transactivator) and *Ccr7*, respectively^18,19^. Cluster 1, showing high expression of *Mmp12* (*Irf4*-specific target gene), was considered a proinflammatory DC based on the enriched expression of *Il1b* and *Csf2rb*^20^. Cluster 2 is expected to regulate M cell maturation via *S100a4* (S100 calcium-binding protein A4)^21^. Clusters 4 and 8 displayed similar gene expression patterns, although cluster 4 showed a higher expression of cyclin genes (*Hist1h1b*, *Birc5*, and *Ube2c*). Cluster 6, which includes *Cd209a* (encoding DC-SIGN)- and *Mgl2* (encoding CD301b)-expressing cells, highly expresses *Cd83*, which can contribute to the generation of regulatory T cells^22^. The features of clusters 7 and 9 were related to antigen processing and presentation, and histone lysine methylation, respectively.

Compared to cDC2 clusters, the LysoDC cluster (cluster 3) displayed a high expression of *Il22ra2* (encoding IL-22 binding protein, IL-22BP), and complement-associated genes, as previously reported^11^. In the PP, IL-22BP can promote antigen uptake by reducing mucus production via the inhibition of IL-22 signaling^23^. Among these clusters, except for proliferative cells (clusters 4 and 8), we thought that clusters 1, 2, and 3 had potential as vaccine adjuvant targets because they were in resting states, which can receive external signals to be activated. Conversely, the other clusters are thought to be activated under steady-state conditions, where tolerance is preferentially maintained^19,24^.

To further analyze the correlation of the profile with the immune system, we visualized the network of immunological functions using the ClueGo plugin application. Both clusters 1 and 2 positively or negatively modulated T cell activation, whereas cluster 3 (LysoDC cluster) was closely related to the induction of chemotaxis, which is a suitable response for changing the tolerogenic to the stimulatory immune environment (Supplementary Fig. 1e and Supplementary Fig. 3). Additionally, it has been reported that LysoDCs stimulated by R848 (TLR7 ligand) or the complement C5a protein could induce antigen-specific CD4^+^ T cell or CD8^+^ T cell responses, respectively^10,25^. Therefore, we focused on cluster 3 as a vaccine adjuvant target, subdivided it into C5aR transcript^+/-^ populations, and analyzed the differentially expressed genes (DEGs) (Supplementary Fig. 1f, g). When the DEGs of C5aR transcript^+^ LysoDCs were compared to those of C5aR transcript^-^ LysoDCs, the biological network of genes highly expressed in C5aR transcript^+^ LysoDCs was closely associated with the regulation of chemotaxis and T cell immunity (Supplementary Fig. 1f, h). Based on this analysis, we expected C5aR^+^ LysoDCs to be suitable target cells for mucosal vaccines (Supplementary Fig. 1i).

### Co1 peptide-mediated C5aR signaling in LysoDCs induces chemotaxis

**Fig. 1:**
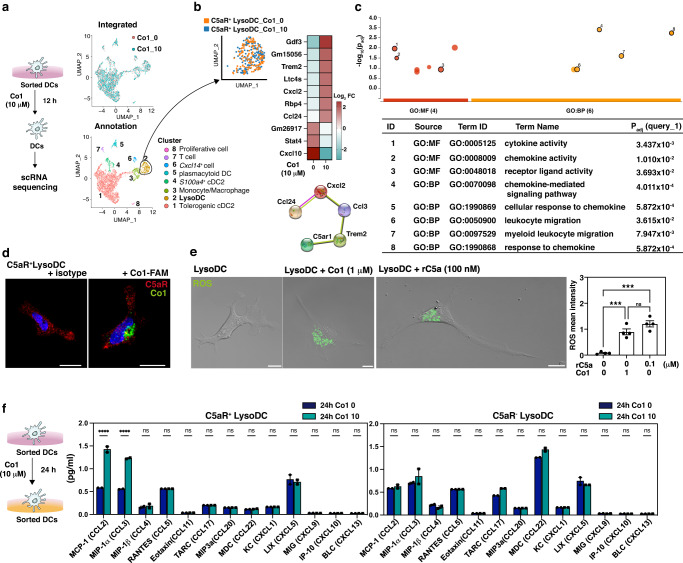
Co1 peptide-mediated C5aR signaling in LysoDCs induces chemotaxis. **a** Single-cell RNA sequencing of CD11c^hi^MHC^hi^SIRPα^+^ Peyer’s patch cells treated with or without the Co1 peptide was performed on 2203 and 2571 individual cells, respectively. UMAP plots show integrated clusters and annotations. **b** UMAP plot showing C5aR^+^ LysoDC (C5aR^+^ LysoDC_Co1_0) and C5aR^+^ LysoDC treated with 10 μM of the Co1 peptide (C5aR^+^ LysoDC_Co1_10) in cluster 2. Heatmap shows the gene expression pattern in C5aR^+^ LysoDC following treatment with the Co1 peptide (10 μM). STRING diagram shows the functional protein-protein interaction network. **c** Manhattan plot shows the gene ontology (GO) enrichment of upregulated genes in C5aR^+^ LysoDC_Co1_10. GO: molecular function (MF), GO: biological process (BP). **d** Sorted LysoDCs show the localization of Co1-FAM (green) in C5aR-expressing cells (red). Scale bars: 10 μm. **e** Live-cell images of sorted C5aR^+^ LysoDCs show ROS generation (green) after treatment with the Co1 peptide or rC5a. Scale bars: 10 μm. Scatterplot represents the mean ROS intensity. **f** Schematic diagram showing the outline of the experiment. The bar graph shows the quantity of each indicated chemokine in the culture supernatants of each indicated cell treated with or without the Co1 peptide. Data are presented as the mean ± standard error (*N* = 3); *p*-values were determined using one-way analysis of variance (ANOVA) followed by Tukey’s multiple comparisons test; ****p < 0.0001.

The conformational features of the Co1 peptide (SFHQLPARSPLP) are similar to those of C5a_65-74_, which is the carboxy-terminal effector region of C5a, a cognate ligand for C5aR (Supplementary Fig. 4a). To investigate whether activation of LysoDCs via Co1 peptide-mediated C5aR signaling can modulate the mucosal microenvironment, CD11c^hi^MHCII^+^SIRPα^+^ PP cells were sorted and stimulated in vitro with 10 μM of the Co1 peptide (Co1_10). Control cells (Co1_0) were incubated in the culture medium only (Supplementary Fig. 4b). After 12 h, the DEGs of each group were generated by scRNA-seq, integrated by anchors and the canonical correlation analysis method, and annotated using cluster biomarkers that were conserved in the two groups (GEO: GSE 212701, Fig. 1a, and Supplementary Fig. 4c). Clusters 1, 4, and 5 were classified as common DC precursor-derived cells due to their expression of *Id2*, while clusters 2 and 3 were classified as monocyte-derived cells due to their expression of *Csf1r* and *Mafb*. The expression pattern of cluster biomarkers in cluster 1 was similar to that of steady-state migratory DCs, which are phenotypically mature and induce Tregs by expressing retinaldehyde dehydrogenase 2 (RALDH2), encoded by *Aldh1a2*^26,27^. This indicates that the maturation of PP cDC2s without stimuli from infection makes it a tolerogenic state. Cluster 4, which highly expressed *S100a4*, was expected to be cluster 2 in Supplementary Fig. 1, and cluster 5, which showed high expression of *Bst2*, was considered plasmacytoid DCs (pDCs). Clusters 2 and 3 represented LysoDCs and monocytes/macrophages, respectively. Among these clusters, cluster 6 (*Cxcl14*^+^ cells), cluster 7 (T cells), and cluster 8 (proliferative cells) were excluded from the subsequent analyses because this study was focused on DCs. Upon stimulation with the Co1 peptide, changes in cell composition and DEGs were not notable in each cluster (Supplemental Fig. 4d, e). However, when C5aR transcript^*+*^ LysoDCs were filtered from cluster 2, and DEGs were compared upon stimulation with the Co1 peptide, we found that upregulated genes were *C5ar*-associated genes containing chemokines (Fig. 1b). Gene ontology for these upregulated genes using gProfiler also showed that the biological functions of these genes were closely related to chemotaxis (Fig. 1c). Based on this analysis, we hypothesized that Co1-mediated C5aR signaling in LysoDCs could contribute to the construction of an immune-stimulatory environment through chemotaxis.

To verify the role of the Co1 peptide in C5aR^+^ LysoDCs, we investigated the localization of 6-carboxyfluorescein-labeled Co1 peptide (Co1-FAM) and the generation of reactive oxygen species (ROS) in sorted C5aR^+^ LysoDCs stimulated with the Co1 peptide (Fig. 1d, e). Given that rC5a-C5aR signaling in LysoDCs induces ROS generation, ROS generation upon Co1 peptide treatment suggests that the Co1 peptide engages C5aR signaling in LysoDCs, which leads to the production of ROS^11^. To confirm the biological role of the Co1 peptide, the level of chemokines was measured in the culture supernatant of Co1 peptide-treated C5aR^+^ or C5aR^-^ LysoDCs (Fig. 1f). The expression levels of CCL2 (MCP-1) and CCL3 (MIP-1α) were significantly increased more than 2-fold in Co1 peptide-treated C5aR^+^ LysoDCs compared to those in C5aR^+^ LysoDCs without Co1 stimulation and in Co1 peptide-treated C5aR^-^ LysoDCs. This finding was also confirmed in vivo.

**Fig. 2:**
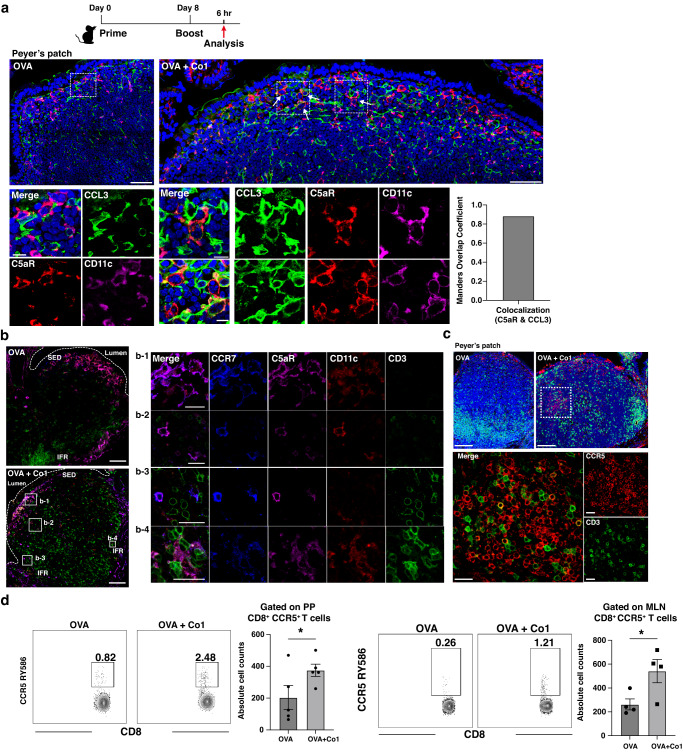
Co1 peptide-mediated C5aR signaling in LysoDCs induces chemotaxis. **a** The distribution of CD11c^+^ (magenta), C5aR^+^ (red), and CCL3^+^ (green) cells in Peyer’s patch (PP) slides prepared from mice immunized according to the schematic diagram, observed using confocal laser scanning microscopy (Scale bars: 50 μm). The section inside the dotted line is enlarged in the below panel (Scale bars: 10 μm). Arrows indicate CCL3-expressing C5aR^+^ LysoDCs. The bar graph shows the colocalization coefficients of C5aR and CCL3. **b** The CCR7^+^ cells were investigated in PP slides prepared from mice immunized according to the schematic diagram (Scale bars: 100 μm). The rectangles are magnified in the right panels (Scale bars: 20 μm). **c** The distribution of CD3^+^ cells (green) and CCR5^+^ cells (red) in PP slides prepared from immunized mice (Scale bars: 100 μm). The section inside the dotted line is magnified in the below panel (Scale bars: 20 μm). **d** The expression of CCR5 in T cells analyzed by flow cytometry in mice immunized with each indicated antigen. The representative contour plot shows the frequency of CCR5^+^ cells in CD3^+^CD8^+^ cells of the PPs and mesenteric lymph nodes (MLNs). The dot plot shows the absolute number of CCR5^+^ cells in CD3^+^CD8^+^ cells of the PPs and MLNs. Data are presented as the mean ± standard error (*N* = 5 mice (PP) and *N* = 4 mice (MLN)). *p*-values were determined using one-way ANOVA followed by Tukey’s multiple comparisons test; **p < 0.005 and ***p < 0.001.

The mice were orally primed and boosted with OVA, either with or without Co1 peptide (OVA group, OVA + Co1 group). Six hours after the final boost, PPs were obtained from the ileum of the small intestine. The PP sections were fixed and stained with each indicated antibody. In the SED of PPs, the expression of CCL3 was primarily observed in some C5aR^-^CD11c^-^ cells in both OVA and OVA + Co1 groups (Fig. 2a). However, as shown in the magnified figures of the area outlined by rectangles, similar to the results obtained for chemokines secreted by Co1 peptide-stimulated C5aR^+^LysoDCs, C5aR^+^ LysoDCs expressing CCL3 were identified in the SED of PPs of the OVA + Co1 group but not the OVA group. Moreover, the colocalization of C5aR and CCL3 was quantitatively analyzed using the Manders Overlap Coefficient (MOC), with a range of MOC values from 0.5 to 1 indicating strong colocalization (Fig. 2a, bar graph). Therefore, we hypothesized that Co1 peptide-mediated C5aR signaling was associated with the induction of CCL3. CCL3 recruits CCR5^+^CD8^+^ T cells to DC-rich regions, leading to efficient priming of the antigen-specific CD8^+^ T cell response^28,29^. Therefore, we examined the expression of chemokine receptor 7 (CCR7) since the expression of CCR7 in LysoDCs can be induced by external signals, such as TLR7 ligands, and CCR7 can promote the migration of LysoDC into the interfollicular region (IFR). As expected, C5aR^+^CD11c^+^ cells in the SED of the OVA + Co1 group exhibited a high level of CCR7 expression and were also observed in the IFR, whereas such cells were not found in the OVA group. This can be observed in the magnified figures of the area outlined by rectangles in Fig. 2b (CCR7 labeled as blue). Thus, we assumed that the recruitment of CCR5^+^ T cells into the PP was promoted. To confirm this, we investigated the distribution of CCR5^+^ cells in the PP of mice immunized with each indicated antigen (Fig. 2c). The localization of CCR5^+^ cells (red), which are outlined with a dotted line and enlarged in the lower panels, was observed in the IFR of the OVA + Co1 group (Fig. 2c). Next, to define these CCR5^+^ cells, cells were prepared from the PPs and mesenteric lymph nodes (MLNs) of each indicated group and analyzed using flow cytometry (Fig. 2d and Supplementary Fig. 5). In the OVA + Co1 group, there was an increase in the number of CD8^+^ T cells expressing CCR5. While numerous CD11c^-^C5aR^-^CCL3^+^ cells were present in the SED of PP, the recruitment of CCR5^+^ cells was specifically observed in the OVA + Co1 group. This finding suggests that the secretion of biologically active CCL3, along with the induction of CCR7 expression in C5aR^+^ LysoDCs, occurs upon oral administration of the Co1 peptide.

### Co1-C5aR signaling induces exogenous antigen cross-presentation in LysoDCs

**Fig. 3:**
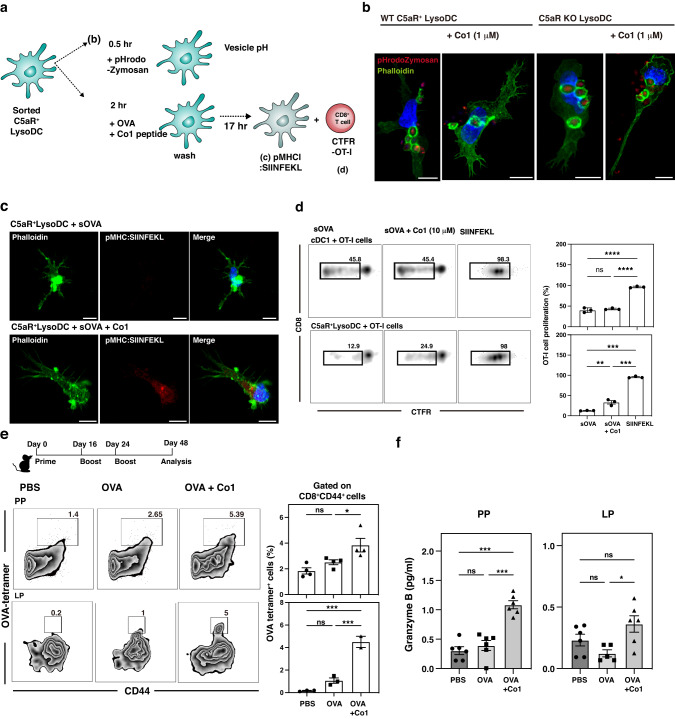
Co1-C5aR signaling induces exogenous antigen cross-presentation in LysoDCs. **a** Schematic diagram showing the experimental outline (**b**–**d**) to identify the function of Co1 peptide signaling in C5aR^+^ LysoDCs. **b,** **c** Confocal microscopy images of sorted cells treated with each indicated molecule showing changes in phagosomal pH using pHrodo-zymosan (red) and phalloidin (green) (**b**) and formation of the pMHCI:SIINFEKL complex using OVA_257-264_ (SIINFEKL) peptide bound to H-2K^b^ monoclonal antibody (red) and phalloidin (green). **c** Nuclei counterstained using Hoechst 33343 (blue). Scale bars: 10 μm. **d** CellTrace^TM^ Far Red (CTFR) dye-labeled OT-1 T cells were co-cultured with cDC1s or C5aR^+^ LysoDCs treated with the indicated molecules. Representative zebra plots showing proliferation of CD8^+^ OT-I T cells measured using flow cytometry after 65 h. Data are presented as the mean ± standard error (*N* = 3). *p*-values were determined using one-way ANOVA followed by Tukey’s multiple comparisons test; **p < 0.01, ***p < 0.001, and ****p < 0.0001. **e**, **f** Seven-week-old female C57BL/6 mice were orally immunized with OVA (50 μg) or OVA (50 μg) plus Co1 (50 μg). Twenty-four days after the final boost, cells were prepared from the PP or lamina propria (LP). **e** Cells analyzed with OVA tetram**e**r, CD3, CD8, and CD44 antibodies using flow cytometry. Bar graphs show the frequency of OVA-specific CD8^+^CD44^+^ cells (*N* = 6 mice). Data are presented as the mean ± standard error (*N* = 3). *p*-values were determined using one-way ANOVA followed by Tukey’s multiple comparisons test; *p < 0.05 and ***p < 0.001. **f** Bar graphs indicating the quantity of granzyme B in the culture supernatant of PP or LP cells co-cultured with SIINFEKL peptide.

The induction of antigen-specific CD8^+^ T cell immune responses after protein-based vaccine application is determined by the function of DCs, for example, exogenous antigen cross-presentation^12^. Given that the activation of C5a-C5aR signaling in LysoDCs elicits antigen cross-presentation through the inhibition of phagosomal acidification via ROS generation, we hypothesized that C5aR signaling stimulated by the Co1 peptide promotes antigen cross-presentation and investigated the sequential events involved in the process (Fig. 3a). Phagosomal pH change was monitored through fluorescence changes in C5aR^+^ LysoDCs fed with Alexa 488-conjugated phalloidin and pH-sensitive (pHrodo) dye-conjugated zymosan (pHrodo-zymosan), which fluoresces at a wavelength of 585 nm at an acidic pH (Fig. 3b). The red particles surrounded by the green ring (phagosome) indicate pHrodo-zymosan in the acidic phagosome. Red fluorescence in phagosomes was observed in C5aR^+^ LysoDCs sorted from wild-type mice (WT C5aR^+^ LysoDC), whereas stimulation with the Co1 peptide did not induce the fluorescence emission of pHrodo-zymosan. In addition, this effect of the Co1 peptide was not observed in LysoDCs sorted from C5aR-knockout mice (C5aR KO LysoDC).

Because the delay in phagosomal acidification was related to the increase in antigen cross-presentation by enhancing antigen exportation to the cytosol, we next observed the formation of MHC class I-loaded OVA_257-264_ SIINFEKL peptide (pMHC:SIINFEKL) complexes in C5aR^+^ LysoDC-fed soluble OVA protein with or without the Co1 peptide (Fig. 3c). This complex (red) was detected only in C5aR^+^ LysoDCs stimulated with OVA and Co1 peptides. To observe the functional correlation between pMHC:SIINFEKL complex formation and antigen-specific CD8^+^ T cell priming, sorted cells were co-cultured with CellTrace^TM^ Far Red dye-labeled CD8^+^ OT-I T cells (Fig. 3d). In the positive control, cDC1s effectively induced OT-I T cell proliferation without additional stimulation because of their intrinsic ability to cross-present exogenous antigens, while C5aR^+^ LysoDCs required stimulation by Co1 peptide to induce OT-I T cell proliferation, suggesting that Co1 peptide stimulation of C5aR^+^ LysoDCs induced cross-priming of CD8^+^ T cells through induction of exogenous OVA cross-presentation. To evaluate the in vivo function of the Co1 peptide for the induction of an antigen-specific CD8^+^ T cell response, mice were orally primed and boosted with each indicated material, and OVA-specific CD8^+^ T cells were identified using an OVA tetramer in cells prepared from the PP and LP at 24 days after the final oral boosting (Fig. 3e). OVA-specific CD8^+^CD44^+^ T cells were increased in the OVA and Co1 peptide (OVA + Co1) group compared to those in the OVA-only group. In addition, when cells were re-stimulated in vitro with the SIINFEKL peptide, they secreted granzyme B (Fig. 3f).

### Oral immunization with the Co1 peptide and recombinant viral antigen evokes an antigen-specific immune response in both the systemic and mucosal area

**Fig. 4:**
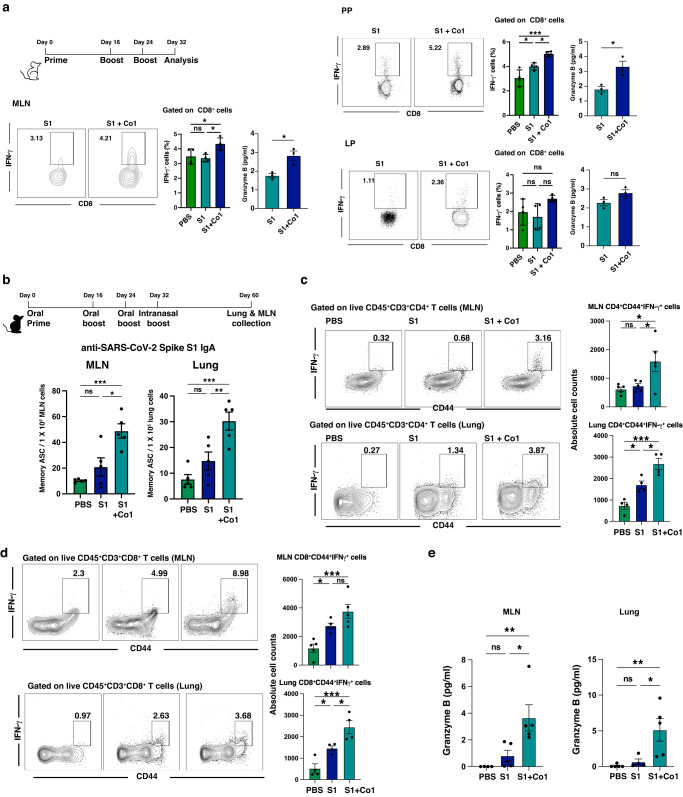
Oral immunization with Co1 peptide and recombinant viral antigen evokes an antigen-specific immune response in both the systemic and mucosal areas. **a** Schematic diagram showing the oral vaccination strategy with S1 or S1 + Co1. Seven-week-old female BALB/c mice were orally immunized with S1 (25 μg) or S1 (25 μg) plus Co1 (50 μg), and the cells were prepared 8 days after the final boost. Representative contour plots indicate the expression level of IFN-γ in live CD45^+^CD3^+^CD8^+^ T cells after in vitro re-stimulation with the SARS-CoV-2 scanning pool for 6 h in each indicated cell (*N* = 6 mice). Bar graphs showing the quantity of granzyme B secreted by re-stimulation with the SARS-CoV-2 S1 scanning pool. **b** Schematic diagram showing the immunization strategy (*N* = 5 mice). Seven-week-old female C57BL/6 mice were immunized with S1 (25 μg) or S1 (25 μg) plus Co1 (50 μg). Representative bar graphs indicate the number of SARS-CoV-2 Spike S1 protein-specific IgA-secreting cells in MLN and lung tissue. **c**, **d** Representative contour plots show the frequency of CD4^+^IFN-γ^+^ T cells (**c**) or CD8^+^IFN-γ^+^ T cells (**d**) in MLN and lung tissue. Bar graphs indicate the absolute cell number of CD4^+^IFN-γ^+^ T cells in MLN and lung tissue. **e** Bar graphs showing the quantity of granzyme B secreted by re-stimulation with PepTivator. *p*-values were determined using one-way ANOVA followed by Tukey’s multiple comparisons test; *p < 0.05, **p < 0.01, ***p < 0.001, and ****p < 0.0001.

Based on the above findings, to evaluate the role of the Co1 peptide as a mucosal adjuvant, the Co1 peptide was applied to the mucosal vaccine model using the S1 domain of the SARS-CoV-2 spike protein as an antigen. The mice were orally primed and boosted with recombinant S1 protein with or without the Co1 peptide. Eight days after the final booster vaccination, cells prepared from each indicated LN and tissue were re-stimulated in vitro with the SARS-CoV-2 S1 scanning pool, consisting of 15-mer length peptides with an 11 amino acid overlap covering the SARS-CoV-2 spike protein. Upon re-stimulation, the frequency of CD8^+^ IFN-γ^+^ T cells and the amount of secreted granzyme B were significantly increased in both MLNs and PPs of mice that were co-administered the S1 and Co1 peptide (S1 + Co1) compared to those administered PBS and S1 alone (Fig. 4a). Since the level of protection against pathogens has shown a correlation with the number of memory CD8^+^ T cells, S1-specific CD8^+^ IFN-γ^+^ T cells were analyzed as memory subsets: memory precursor effector cells (MPEC; CD127^+^KLRG1^-^), TE (transition effectors; CD127^+^KLRG1^+^), EE (early effectors; CD127^-^KLRG1^-^), and SLEC (short-lived effector cells; CD127^-^KLRG1^+^) (Supplementary Fig. 6a). Although a major proportion of S1-specific CD8^+^ IFN-γ^+^ T cells were TE and SLEC, oral immunization with the Co1 peptide induced an increase in MPECs. Additionally, increasing the MPECs by co-administration with the S1 and Co1 peptides was more clearly observed in the LP, where the frequency of S1-specific CD8^+^ IFN-γ^+^ T cells was similar between the S1 and S1 + Co1 groups. Notably, MPECs can develop into long-lived memory cells^30^. To determine whether S1-specific CD8^+^ T cells induced by oral immunization with the Co1 peptide can simultaneously produce the other cytokines, CD8^+^ IFN-γ^+^ T cells were further analyzed (Supplementary Fig. 6b). In the MLNs and PPs, CD8^+^ T cells expressing granzyme B were the major population, but not in the LP. In the S1 + Co1 group, 50% of the CD8^+^ IFN-γ^+^ T cells expressed TNF and IL-2 simultaneously.

Given that systemic priming and intranasal boosting can induce both systemic and respiratory mucosal immunity, we hypothesized that oral immunization and subsequent intranasal boosting could also generate both systemic and respiratory mucosal immunity. Mice were immunized with S1 and S1 + Co1 orally on days 0, 16, and 24, and given booster immunization via the intranasal route on day 32. There was a significant increase in the number of S1-specific IgA-secreting cells in both the lungs and MLNs in the S1 + Co1 group (Fig. 4b). The frequency and absolute cell numbers of the antigen-specific IFN-γ-producing activated/memory CD4^+^ and CD8^+^ T cells (CD4^+^CD44^+^ and CD8^+^CD44^+^ cells, respectively) were also increased in the S1 + Co1 group compared to that of the S1-alone group (Fig. 4c, d). The level of secreted granzyme B upon re-stimulation with S1 peptides was significantly higher in the S1 + Co1 group than that of the S1 group, indicating that intranasal boosting with S1 + Co1 enhances the functionality of the antigen-specific T cells in the lungs and MLNs (Fig. 4e). These data indicate that co-administration with Co1 peptide and antigen can promote the development of antigen-specific memory Th1 cells and CD8^+^ T cells in both systemic and mucosal compartments.

## Discussion

Most currently licensed vaccine adjuvants focus on antibody response induction. However, it is important to develop adjuvants capable of supporting the induction of CD8^+^ T cell responses comparable to those elicited by infection or vaccination with live and/or attenuated viruses. In addition to TLRs, which are generally considered vaccine adjuvant targets, this study showed that complement receptors are potential adjuvant targets capable of promoting chemotaxis and exogenous antigen cross-presentation, resulting in antigen-specific CD8^+^ T cell response induction.

The mucosal immune environment is diverse in structure and function, and the intestinal tract displays regional specialization in immunity through anatomical and physiological differences^31^. Under these diverse circumstances, the mucosal tolerogenic microenvironment is maintained through chemokines by heterogeneous DCs. For instance, CD103^+^ DCs highly express *Ccl22* and induce migration of Treg cells^3,32^. Conversely, the immunomodulatory activity of chemokines can be utilized as effective vaccine adjuvants. The modified vaccinia Ankara vectors are currently being used to develop various vaccines, including a vaccine against SARS-CoV-2 infection, because vaccinia virus infection elicits an antigen-specific CD8^+^ T cell response in a CCL3 and CCL4 chemokine-dependent manner^28,33^. Therefore, we assumed that CCL3 expressed by Co1 peptide-mediated C5aR signaling in LysoDCs may be involved in the effective generation of SARS-CoV-2 S1-specific CD8^+^ T cells.

CCL3, a macrophage inflammatory protein-1α, is mainly secreted by inflammatory cells, and its role is closely related to chemotaxis. In this study, although the upregulation of *Ccl3* gene expression upon treatment with Co1 peptide was not confirmed by scRNA-seq in C5aR^+^ LysoDCs, the expression of CCL3 protein was not only observed in PP tissue sections but was also quantified in the culture supernatant of C5aR^+^ LysoDCs stimulated with the Co1 peptide. This may be related to the post-transcriptional regulation mechanism of *Ccl3* gene expression, such that tristetraprolin (TTP) directly interacts with CCL3 mRNA and then destabilizes CCL3 mRNA^34^. Additionally, we assumed that C5aR^+^ LysoDC stimulated by the Co1 peptide modulates other DCs via CCL3 because CCR1 (a receptor for CCL3) is expressed mainly in immature DCs, including cluster 1, which showed enriched expression of *Ccr1* and *Il12r* (Fig. 1)^35^. DCs activated by antigens and cytokines, such as IL-1, migrate into the MLNs and present antigens to CD4^+^ T cells in the T cell area and induce follicular helper T cells (Tfh)^36^. Although the major role of Tfh is helping B cell proliferation and differentiation of germinal center cells and plasma cells, we focused our interest on the role of Tfh in CD8^+^ T cell modulation, because IL-21 secreted from Tfh was closely related to the regulation of pathogen-specific polyfunctional CD8^+^ effector T cell responses^37^. Therefore, we speculated that cluster 1 cDC2s encounter antigens after being recruited to C5aR^+^ LysoDCs, which were stimulated with the Co1 peptide, migrated to the MLNs, and induced Tfh cells, helping to maintain effector CD8^+^ T cell responses in the systemic immune compartment.

To investigate the adjuvanticity of the Co1 peptide in the mucosal vaccine model, we applied the S1 domain of the SARS-CoV-2 spike protein as an antigen. Although efforts to study vaccines against SARS-CoV-2 infection have mostly focused on protection in the respiratory tract, protection in the gastrointestinal tract is also crucial because this virus has displayed potential for intestinal infection and fecal-oral transmission^38^. In line with a recent study demonstrating the induction of respiratory mucosal immunity through intranasal administration of the spike protein of SARS-CoV-2 after parenteral priming with mRNA-LNP, our results further support this concept^39^. In particular, our results revealed that antigen-specific respiratory mucosal immunity can also be elicited by intranasal co-administration of S1 + Co1 after oral priming and boosting with S1 + Co1. Therefore, we expect that the application of the Co1 peptide will aid in the development of an edible vaccine against SARS-CoV-2.

In summary, our study identified that C5aR^+^ LysoDCs serve as target cells of a mucosal vaccine; their activation via Co1 peptide enhanced vaccination efficiency through the induction of CCL3 expression and exogenous antigen cross-presentation. This postulation was confirmed by the effective generation of antigen-specific CD8^+^ T cells in the SARS-CoV-2 mucosal vaccine model. Therefore, our results have shown that Co1 peptide, a C5aR ligand, is promising as a mucosal vaccine adjuvant while also providing unknown insights into complement receptor signaling as the target of a mucosal vaccine adjuvant.

## Methods

### Mice

Specific pathogen-free seven-week-old female BALB/c and C57BL/6 (B6) mice were purchased from the Koatech Laboratory Center, Korea. OT-I (Thy1.1) mice were gifted by Dr. Kwang Soon Kim (Pohang University of Science and Technology). C5aR knockout mice were purchased from Jackson Laboratory, USA. Mice were maintained according to the protocol of the Animal Center of Jeonbuk National University. All protocols used in this study were approved by the Institutional Animal Care and Use Committee of Jeonbuk National University (Approval No. 2022-056).

### Single-cell RNA sequencing and network analysis

We previously performed gene expression profiling of sorted PP CD11c^hi^MHCclassII^hi^ SIRP^+^ cells (GEO: GSE165040, 33852847). In this study, we re-analyzed the data. The barcode-gene matrices were analyzed using the Seurat 3.1.3 R package, and each cluster’s annotation was defined by the SingleR pipeline using the ImmGen database^40^. To identify biomarkers for each cluster, we compared a single cluster with the others using the Wilcoxon rank sum test (min.pct = 0.25, logfc.threshold = 0.25; only positive ones were filtered) and created a heatmap of the top 20 genes per cluster. Gene-set enrichment tests based on the Gene Ontology DB (category of Biological Process) were conducted with a significant gene list using the g:Profiler tool. Network analyses were performed using CluePedia^41^.

To observe the DEGs in PP CD11c^hi^MHCclassII^hi^ SIRP^+^ cells treated with the Co1 peptide, cells were sorted and cultured in vitro with or without the Co1 peptide in RPMI 1640 supplemented with 10% (w/v) heat-inactivated fetal bovine serum (FBS), colony-stimulating factor 1 (0.1 μg/mL), and colony-stimulating factor 2 (0.5 μg/mL). After 6 h, scRNA-seq was performed by Geninus (Seoul, Korea) according to the protocol provided by 10x Genomics (USA) (GEO: GSE 212701). In the Co1_0 group, 2204 cells were identified with a median of 4.097 genes per cell; in the Co1_10 group, 2571 cells were identified with a median of 3944 genes per cell. For integrative analysis, after a quality check, the datasets were aggregated by canonical correlation analysis of the Seurat R library and normalized by the global-scaling normalization method. To identify conserved cell type markers and DEGs across conditions, the data were analyzed and visualized according to Seurat’s integration tutorial (https://satijalab.org/seurat/articles/integration_introduction.html).

### Immunization experiments

For immunization, C57BL/6 mice were orally administered OVA (50 μg) with or without the Co1 peptide (50 μg) once daily. BALB/c mice or C57BL/6 mice were orally administered Spike S1 (B.1.617.2, Delta Variant, SARS-CoV-2) Avi-His-Tag (25 μg) (BPS Bioscience, USA) together with the Co1 peptide (50 μg) once daily. After two weeks of oral priming with each antigen, the mice were boosted twice a week with each antigen once daily. C57BL/6 mice were orally primed and boosted with each indicated antigen once a day. Eight days after the final oral booster, the mice were intranasally immunized with antigens (20 μg) once daily. At day 60, the mice were euthanized, and the cells were prepared from MLNs and lung tissue.

### Preparation of MLN, PP, and LP cells

The MLNs were harvested and ground on a 50 μm strainer to obtain a single-cell suspension. Ileal PPs were harvested, incubated for 20 min at 37 °C in Hank’s balanced salt solution (HBSS) buffer with 5 mM EDTA and 5% FBS, and then digested for 45 min in RPMI 1640 medium with collagenase D (Roche, 0.5 mg/mL)/DNase I (Sigma, 1 mg/mL) and 10% FBS. For isolated LP cells, small intestine pieces removed with PPs were incubated for 60 min at 37 °C in HBSS buffer with 10 mM EDTA and 5% FBS and then digested with the BD Horizon^TM^ Dri Tumor & Tissue Dissociation Reagent (TTDR, BD Bioscience, USA) according to the manufacturer’s protocol. Dead cells were removed using a dead cell removal kit (Miltenyi Biotec, Germany). To prepare the single cells from the lung tissue, perfusion was performed by injecting ice-cold PBS into the right ventricle of the heart. The lung tissue was then collected, minced, and digested using an enzyme mix from a Lung Dissociation kit (Miltenyi Biotec) according to the manufacturer’s protocol. T cells were enriched using the Pan T cell Isolation Kit II (Miltenyi Biotec).

### Preparation of single cells by cell sorting

To sort the PP CD11c^hi^MHCclassII^hi^ SIRP^+^ cells, PP DCs were enriched using Pan DC MicroBeads (Miltenyi Biotec). Enriched DCs were stained with anti-CD11c (PE/Cy7, BioLegend, USA, 1:50), anti-CD172a (APC/Cy7, BioLegend, 1:100), anti-CD88 (PE, BioLegend, 1:100), anti-I-A/I-E-(BB515, BD Biosciences, 1:200), anti-CD4 (BB700, BD Biosciences, 1:200), and anti-CD317 (APC, Thermo Fisher Scientific, USA, 1:50) antibodies. Cell sorting was performed using FACSAria III (BD Biosciences).

### Immunofluorescence analysis

To observe the interaction between the Co1 peptide and C5aR^+^ LysoDCs, sorted C5aR^+^ LysoDCs were cultured in vitro for 24 h on a chamber slide (Thermo Fisher Scientific). Cultured LysoDCs were treated with the Co1-FAM peptide, fixed in 4% paraformaldehyde (Sigma-Aldrich), and stained with an anti-C5aR antibody (PE, BioLegend, 1:25). To observe CCL3- or CC5-expressing cells in PPs, mice were primed and boosted with OVA + Co1 peptide. Six hours after boosting, PPs were dissected from the terminal ileum of the small intestine. The PPs were frozen in issue-Tek® O.C.T. Compound (Sakura, USA) and sectioned using a cryostat microtome (Thermo Fisher Scientific). After permeabilization with methanol, cryostat sections were blocked with CytoVista^TM^ Blocking Buffer (Thermo Fisher Scientific) and stained with the following antibodies: anti-CCL3 (Thermo Fisher Scientific, 1:50), anti-C5aR (Thermo Fisher Scientific, 1:50), anti-CCR5 (Thermo Fisher Scientific, 1:50), anti-CD11c (Alexa Fluor 647, BioLegend, 1:25), and anti-CD3 (Alexa Fluor 488, BioLegend, 1:25), followed by staining with anti-rabbit IgG (Alexa Fluor Plus 488, Thermo Fisher Scientific, 1:2000) and anti-rat IgG (Alexa Fluor 555, Thermo Fisher Scientific, 1:1000). After counterstaining with Hoechst 33342 (Thermo Fisher Scientific), the samples were mounted using SlowFade Diamond Antifade Mountant (Thermo Fisher Scientific) and observed in Airyscan mode using the Zeiss LSM880 confocal laser scanning microscope (Carl Zeiss). Images were prepared after linear unmixing using the Zen software (Carl Zeiss).

### In vitro ROS assay

Sorted cells cultured in a chamber slide were stimulated with rC5a (100 nM) or the Co1 peptide (1 μM) with the soluble OVA protein for 0.5 h in a culture medium containing the ROS-detection solution (Enzo Life Science, USA), and the fluorescence intensity was monitored in real time using the Zeiss LSM 880 confocal laser scanning microscope (Carl Zeiss, Germany). Data were analyzed using the Zen microscopy software (Carl Zeiss) and GraphPad Prism V9 software (GraphPad Inc., USA).

### Measurement of chemokines

The sorted C5aR^+^ LysoDCs or C5aR^-^ LysoDCs were stimulated with Co1 peptide (10 μM) for 24 h. The number of chemokines in the culture supernatant was quantified using the LEGENDplex^TM^ Mouse Proinflammatory Chemokine Panel (BioLegend) according to the manufacturer’s instructions. Data were analyzed using GraphPad Prism V9 software.

### Flow cytometry analysis

To further investigate the recruitment of CCR5^+^ cells, BALB/c mice were orally immunized, as described above. Live cells were distinguished using the BD Horizon™ Fixable Viability Stain (FVS) Reagents 510 (BD Biosciences). The surface markers of cells prepared from the MLNs and PPs of immunized mice were stained with anti-CD45 (BV605, BD Biosciences, 1:100), anti-CD19 (BB515, BD Biosciences, 1:100), anti-CD3 (BV421, BD Biosciences, 1:100), anti-CD8 (APC, BD Biosciences, 1:100), anti-CD4 (BV700, BD Biosciences, 1:100), and anti-CCR5 (RY586, BD Biosciences, 1:50) antibodies.

To analyze OVA-specific CD8^+^ T cells, C57BL/6 mice were orally primed and boosted with OVA with or without the Co1 peptide. At 24 days after the final boost, cells were prepared from the PP and LP as mentioned above and then stained with T-Select I-A^d^ OVA323-339 Tetramer-APC (MBL, Japan) according to the manufacturer’s instructions, followed by staining with anti-CD3 (PerCP-Cy5.5, BioLegend, 1:100), anti-CD8 (FITC, MBL, 1:50), and anti-CD44 (PE, BioLegend, 1:50) antibodies.

BALB/c mice were orally primed and boosted to analyze the S1 domain of SARS-CoV-2 specific CD8^+^ T cells. Ten days after the final boost, the cells were prepared from the MLN, PP, and LP, as mentioned above. For in vitro re-stimulation, the cells were cultured in media containing the SARS-CoV-2 S1 scanning pool (Mabtech, Sweden), brefeldin A, and monensin (Thermo Fisher Scientific). After 8 h, live cells were distinguished using BD Horizon™ Fixable Viability Stain (FVS) Reagents 510, and the surface markers were stained with anti-CD45 (APC-Cy7, BD Biosciences, 1:100), anti-CD3 (Alexa Fluor 700, Thermo Fisher Scientific, 1:100), anti-CD4 (PerCP-Cy5.5, BioLegend, 1:100), anti-CD8 (Super bright 436, BD Biosciences, 1:100), anti-KLRG1 (FITC, BioLegend, 1:50), and anti-CD127 (BV711, BD Biosciences, 1:50) antibodies. For intracellular cytokine staining, cells were fixed and permeabilized with FoxP3 staining buffer (Thermo Fisher Scientific) and then labeled with the following antibodies: anti-TNF (APC, Thermo Fisher Scientific, 1:50), anti-granzyme B (PE, Thermo Fisher Scientific, 1:50), anti-IFN-γ (PE-CF594, BD Biosciences, 1:50), and anti-IL-2 (PE-Cy7, Thermo Fisher Scientific, 1:50).

The enriched T cells prepared from orally and intranasally immunized C57BL/6 mice were co-cultured with T cell depleted splenocytes, PepTivator® SARS-CoV-2 Prot S Complete (Miltenyi Biotec), and a protein transport inhibitor cocktail (Thermo Fisher Scientific). After 8 h, dead cells were stained using BD Horizon™ Fixable Viability Stain (FVS) Reagents 510, and the surface markers were stained using anti-CD45 (BV605, BD Biosciences, 1:100), anti-CD3 (Alexa Fluor 488, Thermo Fisher Scientific, 1:100), anti-CD4 (BB700, BD Biosciences, 1:100), and anti-CD44 (APC-Cy7, BD Biosciences, 1:100) antibodies. After fixing and permeabilization, cells were labeled with anti-IFN-γ (APC, Biolegend, 1:50) antibody.

Labeled cells were acquired using a BD FACSymphony (BD Biosciences), and data were analyzed using the BD FlowJo v10 software (BD Biosciences) and GraphPad Prism V9 software.

### Analysis of phagosomal antigen degradation in C5aR^+^LysoDCs

To monitor changes in the phagosome pH via stimulation with the Co1 peptide, sorted cells were co-treated with pHrodo Red Zymosan Bioparticle Conjugate and Alexa 488-conjugated phalloidin (Thermo Fisher Scientific) during stimulation with the Co1 peptide. To test exogenous antigen cross-presentation, sorted cells treated with the OVA + Co1 peptide for 2 h were vigorously washed and further cultured in vitro for 17 h. After fixation, the cells were labeled with APC-conjugated anti-SIINFEKL peptides bound to H-2K^b^ monoclonal antibodies and Alexa 488-conjugated phalloidin and counterstained with Hoechst 33342. The fluorescence intensity was monitored in Airyscan mode using the Zeiss LSM 880 confocal laser scanning microscope (Carl Zeiss).

### In vitro T cell assays

Sorted cells treated with sOVA were stimulated with or without the Co1 peptide for 2 h and then vigorously washed and co-cultured with CTFR (Thermo Fisher Scientific) dye-labeled CD8^+^ OT-I T cells. After 3 days, the decrease in CTFR signal in Thy 1.1^+^CD8^+^ T cells was monitored using flow cytometry.

### Measurement of granzyme B

To quantify granzyme B secreted by antigen-specific T cells, cells were prepared from PP, LP, MLN, or lung tissue of immunized BALB/c or C57BL/6 mice, and then co-cultured with the SIINFEKL peptide (InvivoGen), SARS-CoV-2 S1 scanning pool (Mabtech), or PepTivator® SARS-CoV-2 Prot S Complete (Miltenyi Biotec) for 24 h. The quantity of granzyme B in the culture media was quantified using the Granzyme B Mouse ProQuantum Immunoassay Kit (Thermo Fisher Scientific) according to the manufacturer’s instructions.

### Measurement of IgA-secreting cells

To evaluate the number of SARS-CoV-2 S1-specific IgA-secreting memory cells, prepared cells were cultured with R848 (1 μg/ml) and rmIL-2 (10 ng/ml) in a culture plate for 48 h according to the manufacturer’s instructions (Mabtech). The cells were washed, resuspended in RPMI 1640 medium containing 10% heat-inactivated FBS, and added to an enzyme-linked immunosorbent spot (ELISpot) plate coated with recombinant SARS-CoV-2 S1 protein. The cells were incubated for 24 h and then removed from the ELISpot plate. Alkaline phosphate-conjugated anti-mouse IgA antibody was incubated in the ELISpot plate for 2 h, and the spots were developed using BCIP/NBT.

### Statistical analysis

The number of mice used in each experiment is shown in the figure legends. Quantitative data are shown as the mean ± standard error of the mean. The *p*-values were determined using one-way analysis of variance (ANOVA) followed by Tukey’s multiple comparisons test, and statistical tests were performed using the GraphPad Prism V9 software.

### Reporting summary

Further information on research design is available in the Nature Research Reporting Summary linked to this article.

### Supplementary information

Reporting Summary

Supplementary Figures

## Data Availability

All data needed to evaluate the conclusions in the paper are present in the paper and the Supplementary Materials. The scRNA-seq data analyzed in this study can be obtained from the NCBI Gene Expression Omnibus (GEO) (accession number GSE 212701).
