# Supplementary material for: C5aR+ dendritic cells fine-tune the Peyer’s patch microenvironment to induce antigen-specific CD8+ T cells
Source: NPJ Vaccines. 2023 Aug 14;8:120. doi: 10.1038/s41541-023-00720-z (PMC10425327; doi:10.1038/s41541-023-00720-z)
Supplement: Supplementary file 1 — Reporting Summary [file 41541_2023_720_MOESM1_ESM.pdf]

nature portfolio

Corresponding author(s): Yong-Suk Jang

Last updated by author(s): Jun 29, 2023

Reporting Summary

Nature Portfolio wishes to improve the reproducibility of the work that we publish. This form provides structure for consistency and transparency in reporting. For further information on Nature Portfolio policies, see our Editorial Policies and the Editorial Policy Checklist.

Statistics

For all statistical analyses, confirm that the following items are present in the figure legend, table legend, main text, or Methods section.

n/a	Confirmed	
☐	☒	The exact sample size (*n*) for each experimental group/condition, given as a discrete number and unit of measurement
☒	☐	A statement on whether measurements were taken from distinct samples or whether the same sample was measured repeatedly
☐	☒	The statistical test(s) used AND whether they are one- or two-sided<br>*Only common tests should be described solely by name; describe more complex techniques in the Methods section.*
☐	☒	A description of all covariates tested
☐	☒	A description of any assumptions or corrections, such as tests of normality and adjustment for multiple comparisons
☐	☒	A full description of the statistical parameters including central tendency (e.g. means) or other basic estimates (e.g. regression coefficient) AND variation (e.g. standard deviation) or associated estimates of uncertainty (e.g. confidence intervals)
☐	☒	For null hypothesis testing, the test statistic (e.g. *F*, *t*, *r*) with confidence intervals, effect sizes, degrees of freedom and *P* value noted<br>*Give P values as exact values whenever suitable.*
☒	☐	For Bayesian analysis, information on the choice of priors and Markov chain Monte Carlo settings
☒	☐	For hierarchical and complex designs, identification of the appropriate level for tests and full reporting of outcomes
☒	☐	Estimates of effect sizes (e.g. Cohen's *d*, Pearson's *r*), indicating how they were calculated

Our web collection on statistics for biologists contains articles on many of the points above.

Software and code

Policy information about availability of computer code

Data collection	BD FACSymphony (BD Biosciences), Zen software (Carl Zeiss),CFX Maestro Software
Data analysis	BD FlowJo v10 software (BD Biosciences), GraphPad Prism V9, Zen software (Carl Zeiss).

For manuscripts utilizing custom algorithms or software that are central to the research but not yet described in published literature, software must be made available to editors and reviewers. We strongly encourage code deposition in a community repository (e.g. GitHub). See the Nature Portfolio guidelines for submitting code & software for further information.

Data

Policy information about availability of data

All manuscripts must include a data availability statement. This statement should provide the following information, where applicable:

- Accession codes, unique identifiers, or web links for publicly available datasets
- A description of any restrictions on data availability
- For clinical datasets or third party data, please ensure that the statement adheres to our policy

Data supporting the finding of this study can be obtained from the NCBI GEO (accession number GSE 212701.

Research involving human participants, their data, or biological material

Policy information about studies with human participants or human data. See also policy information about sex, gender (identity/presentation), and sexual orientation and race, ethnicity and racism.

Reporting on sex and gender	*Use the terms sex (biological attribute) and gender (shaped by social and cultural circumstances) carefully in order to avoid confusing both terms. Indicate if findings apply to only one sex or gender; describe whether sex and gender were considered in study design; whether sex and/or gender was determined based on self-reporting or assigned and methods used. Provide in the source data disaggregated sex and gender data, where this information has been collected, and if consent has been obtained for sharing of individual-level data; provide overall numbers in this Reporting Summary. Please state if this information has not been collected. Report sex- and gender-based analyses where performed, justify reasons for lack of sex- and gender-based analysis.*
Reporting on race, ethnicity, or other socially relevant groupings	*Please specify the socially constructed or socially relevant categorization variable(s) used in your manuscript and explain why they were used. Please note that such variables should not be used as proxies for other socially constructed/relevant variables (for example, race or ethnicity should not be used as a proxy for socioeconomic status). Provide clear definitions of the relevant terms used, how they were provided (by the participants/respondents, the researchers, or third parties), and the method(s) used to classify people into the different categories (e.g. self-report, census or administrative data, social media data, etc.) Please provide details about how you controlled for confounding variables in your analyses.*
Population characteristics	*Describe the covariate-relevant population characteristics of the human research participants (e.g. age, genotypic information, past and current diagnosis and treatment categories). If you filled out the behavioural & social sciences study design questions and have nothing to add here, write "See above."*
Recruitment	*Describe how participants were recruited. Outline any potential self-selection bias or other biases that may be present and how these are likely to impact results.*
Ethics oversight	*Identify the organization(s) that approved the study protocol.*

Note that full information on the approval of the study protocol must also be provided in the manuscript.

Field-specific reporting

Please select the one below that is the best fit for your research. If you are not sure, read the appropriate sections before making your selection.

☒ Life sciences ☐ Behavioural & social sciences ☐ Ecological, evolutionary & environmental sciences

For a reference copy of the document with all sections, see nature.com/documents/nr-reporting-summary-flat.pdf

Life sciences study design

All studies must disclose on these points even when the disclosure is negative.

Sample size	We have minimized both the number of animals per group and the number of experimental groups in order to compare the antigen-specific immune response.
Data exclusions	No data were excluded.
Replication	For the analysis of cells expressing chemokines in tissue slides, more than 3 slides were analyzed, and representative pictures were presented. Analysis of the antigen-specific immune response was repeated three times, and representative results are presented.
Randomization	The mice were randomly allocated to groups.
Blinding	Experiments were performed by displaying numeric ID tags that do not represent specific treatment groups.

Reporting for specific materials, systems and methods

We require information from authors about some types of materials, experimental systems and methods used in many studies. Here, indicate whether each material, system or method listed is relevant to your study. If you are not sure if a list item applies to your research, read the appropriate section before selecting a response.

Materials & experimental systems

n/a	Involved in the study
	☒ Antibodies
☒	☐ Eukaryotic cell lines
☒	☐ Palaeontology and archaeology
	☒ Animals and other organisms
☒	☐ Clinical data
☒	☐ Dual use research of concern
☒	☐ Plants

Methods

n/a	Involved in the study
☒	☐ ChIP-seq
	☒ Flow cytometry
☒	☐ MRI-based neuroimaging

Antibodies

Antibodies used	anti-CD45 (BV605, BD Biosciences, 1:100), anti-CD19 (BB515, BD Biosciences, 1:100), anti-CD3 (BV421, BD Biosciences, 1:100), anti-anti-CD3 (PerCP-Cy5.5, BioLegend, 1:100), anti-CD8 (FITC, MBL, 1:50), and anti-CD44 (PE, BioLegend, 1:50) CD8 (APC, BD Biosciences, 1:100), anti-CD4 (BV700, BD Biosciences, 1:100), and anti-CCR5 (RY586, BD Biosciences, 1:50) antibodies, anti-CD45 (APC-Cy7, BD Biosciences, 1:100), anti-CD3 (Alexa Fluor 700, Thermo Fisher Scientific, 1:100), anti-CD4 (PerCP-Cy5.5, BioLegend, 1:100), anti-CD8 (Super bright 436, BD Biosciences, 1:100), anti-KLRG1 (FITC, BioLegend, 1:50), and anti-CD127 (BV711, BD Biosciences, 1:50) antibodies. anti-TNF (APC, Thermo Fisher Scientific, 1:50), anti-granzyme B (PE, Thermo Fisher Scientific, 1:50), anti-IFN-γ (PE-CF594, BD Biosciences, 1:50), and anti-IL-2 (PE-Cy7, Thermo Fisher Scientific, 1:50). anti-CD45 (BV605, BD Biosciences, 1:100), anti-CD3 (Alexa Fluor 488, Thermo Fisher Scientific, 1:100), anti-CD4 (BB700, BD Biosciences, 1:100), and anti-CD44 (APC-Cy7, BD Biosciences, 1:100), anti-IFN-γ (APC, Biolegend, 1:50) antibody, anti-CCL3 (Thermo Fisher Scientific, 1:50), anti-C5aR (Thermo Fisher Scientific, 1:50), anti-CCR5 (Thermo Fisher Scientific, 1:50), anti-CD11c (Alexa Fluor 647, BioLegend, 1:25), and anti-CD3 (Alexa Fluor 488, BioLegend, 1:25), followed by staining with anti-rabbit IgG (Alexa Fluor Plus 488, Thermo Fisher Scientific, 1:2000) and anti-rat IgG (Alexa Fluor 555, Thermo Fisher Scientific, 1:1000). Enriched DCs were stained with anti-CD11c (PE/Cy7, BioLegend, USA, 1:50), anti-CD172a (APC/Cy7, BioLegend, 1:100), anti-CD88 (PE, BioLegend, 1:100), anti-I-A/I-E-(BB515, BD Biosciences, 1:200), anti-CD4 (BB700, BD Biosciences, 1:200), and anti-CD317 (APC, Thermo Fisher Scientific, USA, 1:50) antibodies.
Validation	Antibodies were titrated prior to use in each assay.

Animals and other research organisms

Policy information about studies involving animals; ARRIVE guidelines recommended for reporting animal research, and Sex and Gender in Research

Laboratory animals	OT-I (Thy1.1) mice were gifted by Dr. Kwang Soon Kim (Pohang University of Science and Technology).<br>Specific pathogen-free seven-week-old female BALB/c and C57BL/6 (B6) mice were purchased from the Koatech Laboratory Center, Korea.<br>C5aR knockout mice were purchased from the Jackson Laboratory, USA.
Wild animals	We did not use wild animals.
Reporting on sex	All findings apply to female mice.
Field-collected samples	We did not use field-collected samples.
Ethics oversight	Mice were maintained according to the protocol of the Animal Center of the Jeonbuk National University. All protocols used in this study were approved by the Institutional Animal Care and Use Committee of Jeonbuk National University (Approval No.2022-056).

Note that full information on the approval of the study protocol must also be provided in the manuscript.

Flow Cytometry

Plots

Confirm that:

☒ The axis labels state the marker and fluorochrome used (e.g. CD4-FITC).

☐ The axis scales are clearly visible. Include numbers along axes only for bottom left plot of group (a 'group' is an analysis of identical markers).

☒ All plots are contour plots with outliers or pseudocolor plots.

☒ A numerical value for number of cells or percentage (with statistics) is provided.

Methodology

Sample preparation	MLNs were harvested and ground on a 50 µm strainer to obtain a single-cell suspension. Ileal PPs were harvested, incubated for 20 min at 37 °C in Hank's balanced salt solution (HBSS) buffer with 5 mM EDTA and 5% FBS, and then digested for 45 min

	in RPMI 1640 medium with collagenase D (Roche, 0.5 mg/mL)/DNase I (Sigma, 1 mg/mL) and 10% FBS as previously described11. For isolated LP cells, small intestine pieces removed with PPs were incubated for 60 min at 37 °C in HBSS buffer with 10 mM EDTA and 5% FBS and then digested with the BD HorizonTM Dri Tumor & Tissue Dissociation Reagent (TTDR, BD Bioscience, USA) according to the manufacturer's protocol. Dead cells were removed using the dead cell removal kit (Miltenyi Biotec, Germany). To prepare the single cells from the lung tissue, perfusion was performed by injecting ice-cold PBS into the right ventricle of the heart, and then the lung tissue was collected, minced, and digested using an enzyme mix from a Lung Dissociation kit (Miltenyi Biotec) according to the manufacturer's protocol. T cells were enriched using a Pan T cell Isolation kit II (Miltenyi Biotec).
Instrument	BD FACSymphony (BD Biosciences)
Software	BD FlowJo v10 software (BD Biosciences)
Cell population abundance	N/A
Gating strategy	1) FSC vs SSC (lymohocytes and singlet), 2) FVS 510 - cells (live cells) 3)CD45+CD3+ cells (T cells)

☒ Tick this box to confirm that a figure exemplifying the gating strategy is provided in the Supplementary Information.
