# Supplementary material for: C5aR+ dendritic cells fine-tune the Peyer’s patch microenvironment to induce antigen-specific CD8+ T cells
Source: NPJ Vaccines. 2023 Aug 14;8:120. doi: 10.1038/s41541-023-00720-z (PMC10425327; doi:10.1038/s41541-023-00720-z)
Supplement: Supplementary file 2 — Supplementary Figures [file 41541_2023_720_MOESM2_ESM.pdf]

Supplementary Figure 1

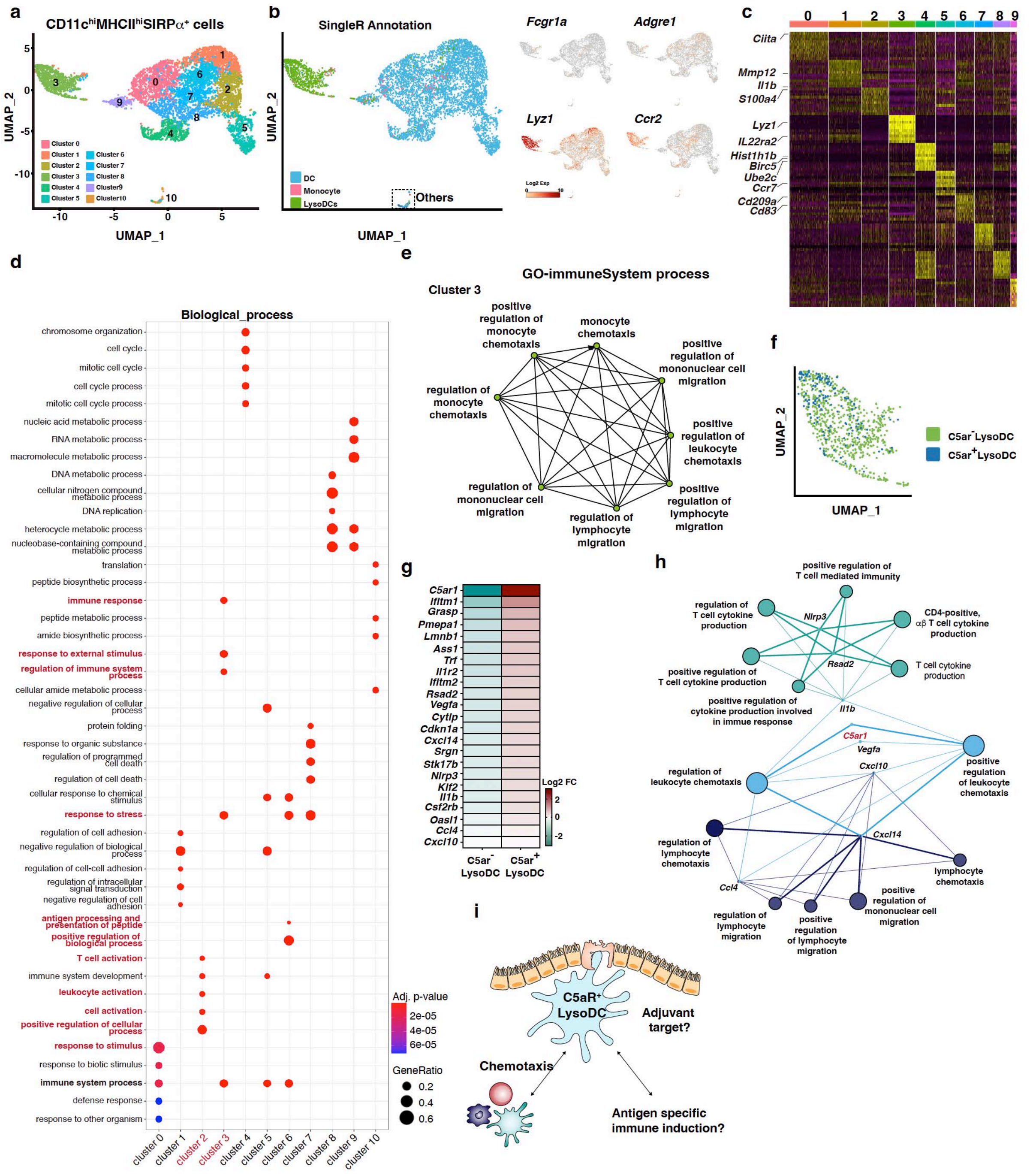

Supplementary Figure 1. C5aR^{+} LysoDCs are an attractive target for mucosal vaccine delivery. Single-cell RNA sequencing (scRNA-seq) of sorted CD11c^{hi}MHC^{hi}SIRPα^{+} Peyer's patch cells was performed on 7,463 individual cells. **a** High-resolution uniform manifold approximation and projection (UMAP) plot defining 11 clusters. **b** scRNA-seq data were annotated by SingleR based on Immgen's reference dataset. The expression pattern of each gene visualized using a feature plot. **c** Heatmap showing the top 20 most differentially expressed genes (DEGs) in each indicated cluster. **d** Dot plot showing the most significant Gene Ontology (GO) terms in each cluster. GO terms in each cluster were chosen from the GO_stat results if their adjusted *p*-value (adj. *p*-value) < 0.05. The dot size was determined using GeneRatio. **e** Network diagram showing the enriched GO-immune system processes

predicted from a list of 2-fold upregulated genes in cluster 3 compared to other clusters. **f** UMAP plot showing C5aR$^+$ LysoDCs and C5aR$^-$ LysoDCs in cluster 3. **g** Heatmap showing the DEGs between C5aR$^+$ LysoDCs and C5aR$^-$ LysoDCs. **h** Network diagram showing the relationship between the GO-immune system processes and genes upregulated in C5aR$^+$ LysoDCs compared toC5aR$^-$ LysoDCs. **i** Schematic showing our hypothesis: the potential of C5aR$^+$ LysoDCs as a target of mucosal vaccines.

Supplementary Figure 2

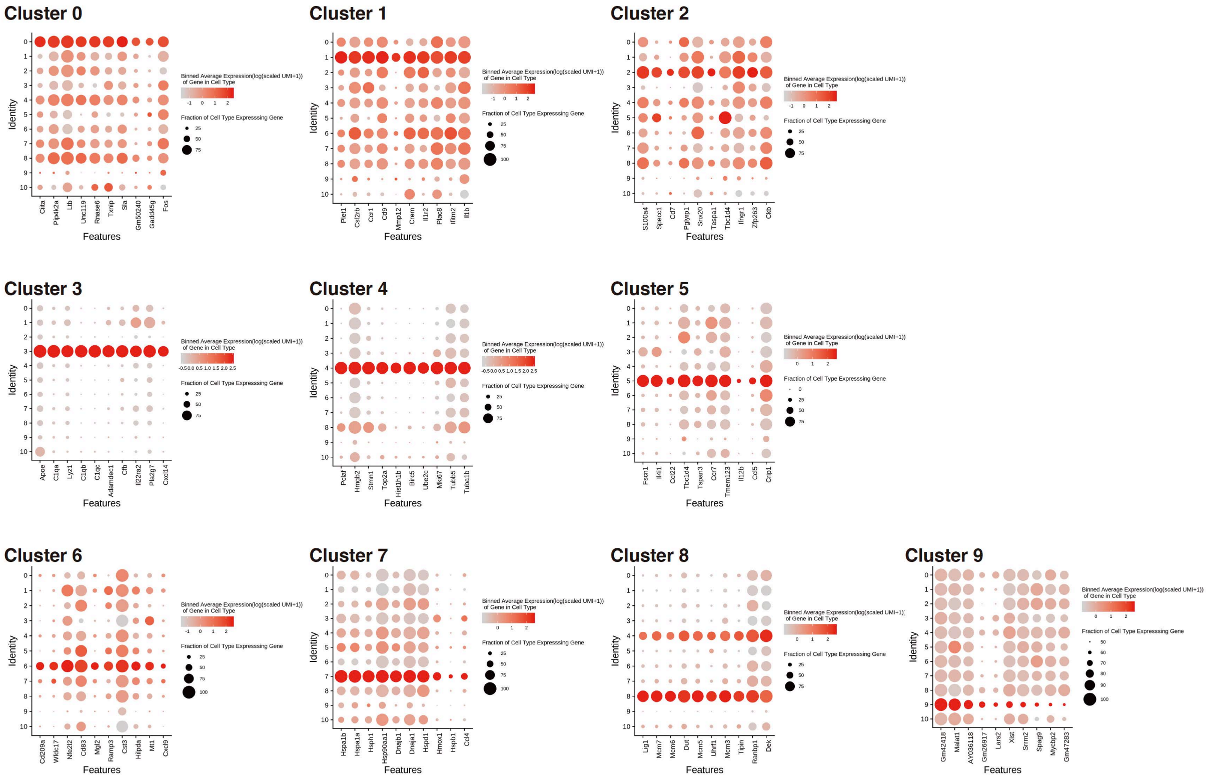

Supplementary Figure 2. Each cluster of CD11c^{hi}MHC^{hi}SIRPα^{+} Peyer's patch cells have a differentially expressed gene-set. Dot plot showing the top 10 most differentially expressed genes in each cluster

Supplementary Figure 3

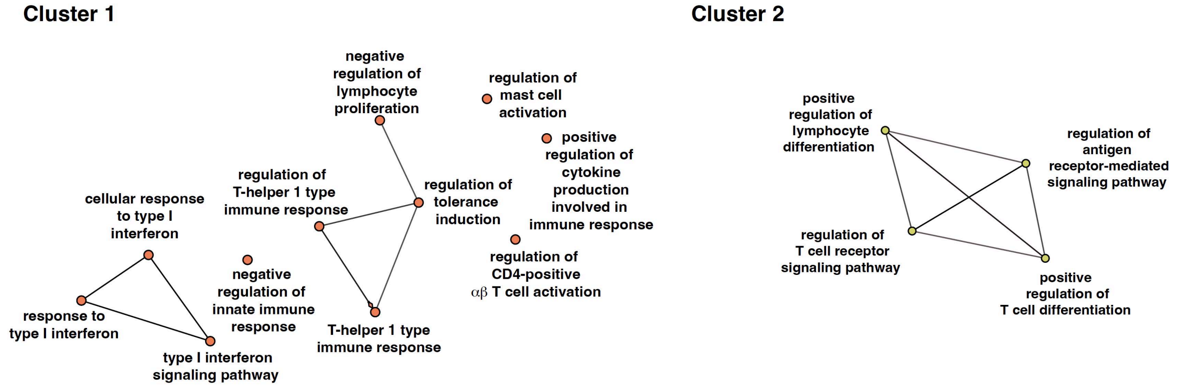

Supplementary Figure 3. The immunological function network shows the relation between the clusters and T cell activation. Network diagram showing the enriched gene ontology-immune system processes predicted from a list of 2-fold upregulated genes in each indicated cluster compared to other clusters**.**

Supplementary Figure 4

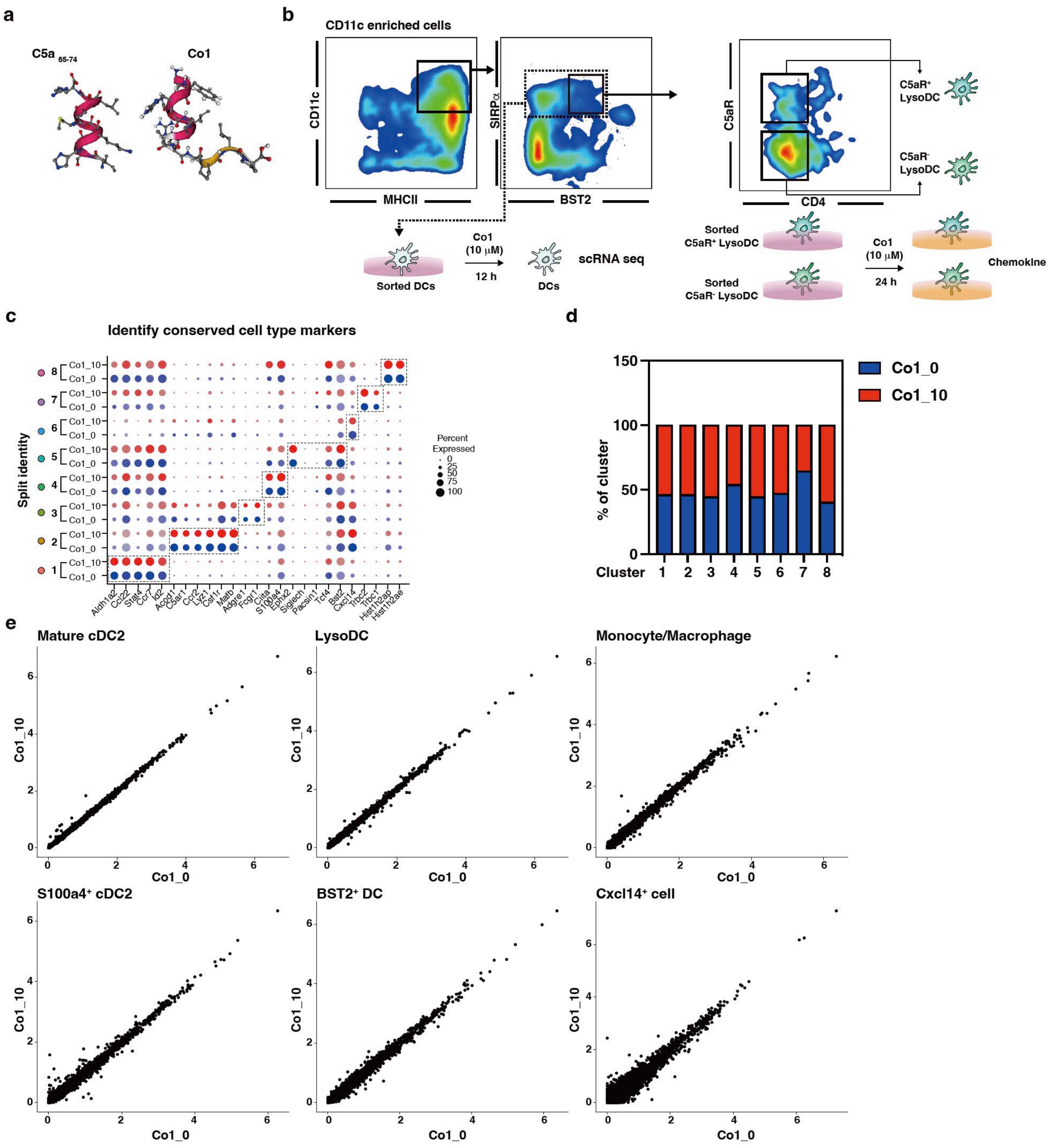

Supplementary Figure 4. Co1 peptide-mediated C5aR signaling in LysoDC can modulate the mucosal immunogenic environment through chemotaxis. a Conformational features of the Co1 peptide and $C5a_{65-74}$ predicted by PEP-FOLD3. **b** Flow cytometry plots showing the sorting strategy for $C5aR^+$ LysoDCs or $C5aR^-$ LysoDCs in CD11c-enriched Peyer's patch cells. **c** Dot plot displaying conserved cell type markers in each indicated condition and cluster. **d** Plot showing the cell proportion in each cell cluster identified in Fig. 1A. **e** Scatter plots showing the differences induced by stimulation with the Co1 peptide in each cluster.

Supplementary Figure 5

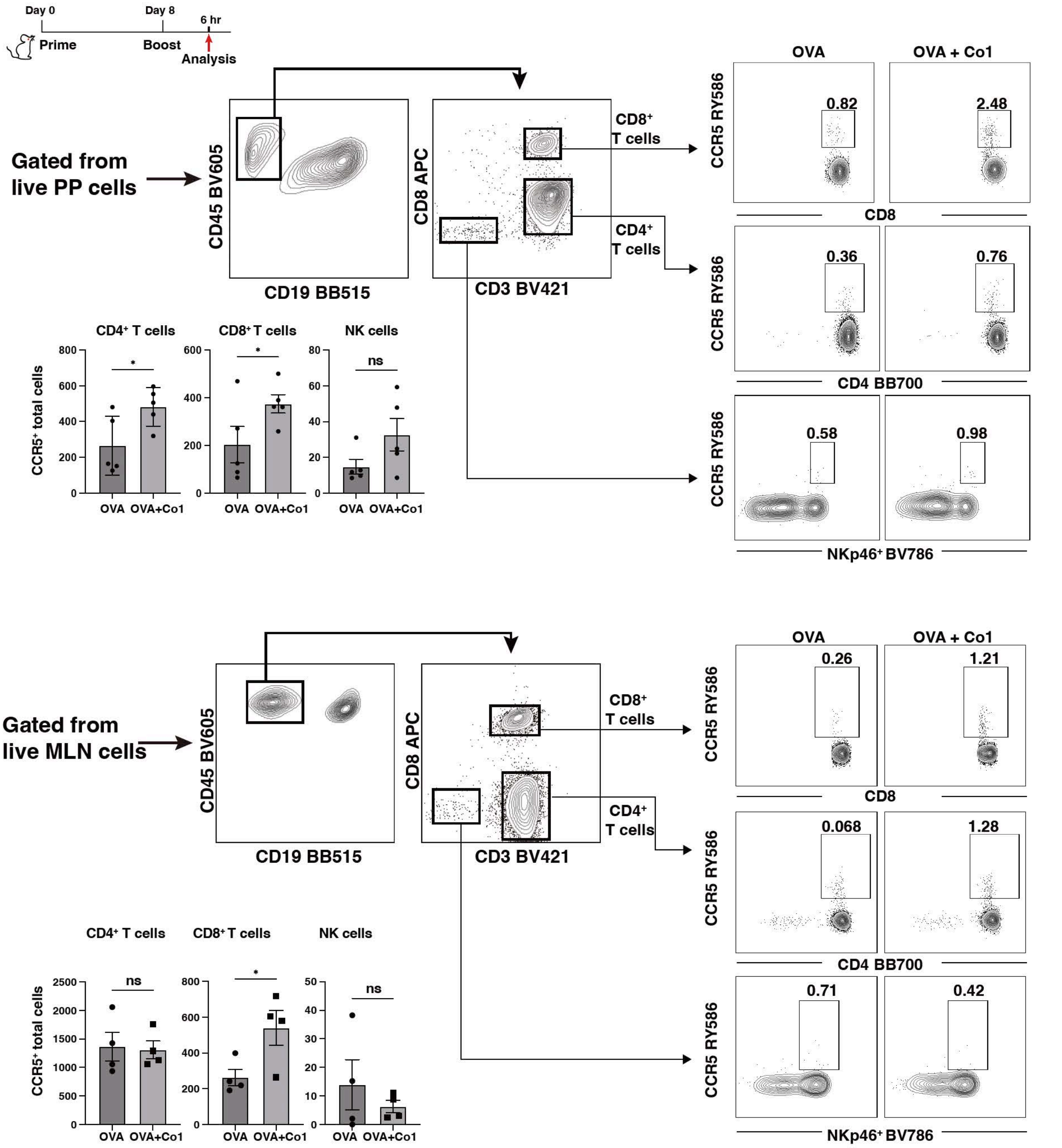

Supplementary Figure 5. Co1 peptide-mediated C5aR signaling in LysoDC can modulate the mucosal immunogenic environment through chemotaxis. To characterize s1$^+$ cells, mice were immunized using each indicated antigen as shown in the schematic diagram. Peyer's patch (PP) or mesenteric lymph node (MLN) cells in mice were prepared and analyzed by flow cytometry. Contour plots and summary columns are presented (N = 4). Data are presented as the mean ± standard error (N = 3). p-values were determined using one-way analysis of variance (ANOVA) followed by Tukey multiple comparisons test, $^{**}p < 0.005$, $^{***}p < 0.001$.

Supplementary Figure 6

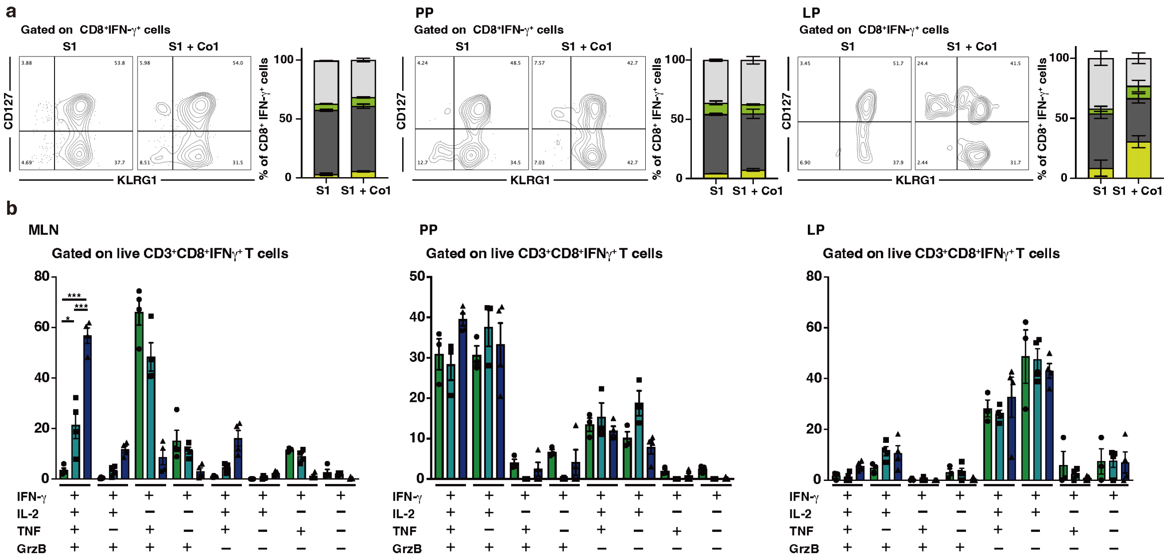

Supplementary Figure 6. Oral immunization of the Co1 peptide with recombinant viral protein elicits an antigen-specific CD8$^+$ T cell response. a Representative contour plots showing the expression levels of CD127 and KLRG1on CD3$^+$CD8$^+$ IFN-γ$^+$ T cells after in vitro re-stimulation with the SARS-CoV-2 scanning pool for 6 h in each indicated cell. **b** Representative bar graphs summarizing the frequency of SARS-CoV-2 specific CD8$^+$ T cells expressing each cytokine (N = 6 mice), *p*-values were determined using one-way analysis of variance (ANOVA) followed by Tukey multiple comparisons test, *$p < 0.05$, **$p < 0.01$, ***$p < 0.001$, ****$p < 0.0001$. LP, lamina propria.
